# The Dual Role of Antimicrobial Peptides in Autoimmunity

**DOI:** 10.3389/fimmu.2020.02077

**Published:** 2020-09-02

**Authors:** Wenjie Liang, Julien Diana

**Affiliations:** Centre National de la Recherche Scientifique (CNRS), Institut Necker Enfants Malades, Institut National de la Santé et de la Recherche Médicale (INSERM), Université de Paris, Paris, France

**Keywords:** autoimmune diseases, antimicrobial host defense peptides, innate immunity, defensin, cathelicidin

## Abstract

Autoimmune diseases (AiDs) are characterized by the destruction of host tissues by the host immune system. The etiology of AiDs is complex, with the implication of multiple genetic defects and various environmental factors (pathogens, antibiotic use, pollutants, stress, and diet). The interaction between these two compartments results in the rupture of tolerance against self-antigens and the unwanted activation of the immune system. Thanks to animal models, the immunopathology of many AiDs is well described, with the implication of both the innate and adaptive immune systems. This progress toward the understanding of AiDs led to several therapies tested in patients. However, the results from these clinical trials have not been satisfactory, from reversing the course of AiDs to preventing them. The need for a cure has prompted many investigators to explore alternative aspects in the immunopathology of these diseases. Among these new aspects, the role of antimicrobial host defense peptides (AMPs) is growing. Indeed, beyond their antimicrobial activity, AMPs are potent immunomodulatory molecules and consequently are implicated in the development of numerous AiDs. Importantly, according to the disease considered, AMPs appear to play a dual role in autoimmunity with either anti- or pro-inflammatory abilities. Here, we aimed to summarize the current knowledge about the role of AMPs in the development of AiDs and attempt to provide some hypotheses explaining their dual role. Definitely, a complete understanding of this aspect is mandatory before the design of AMP-based therapies against AiDs.

## Introduction

Autoimmune diseases (AiDs) have been originally characterized by the destruction of a specific host cell type by autoantigen-specific T and B cells. A higher frequency of autoreactive T cells circulates in the body of autoimmune-prone individuals due to a defect in the thymic selection. Additionally, a defect in the peripheral tolerance allows the activation of the autoreactive lymphocytes and the subsequent destruction of the host cells. This terminal step of the immunopathology of AiDs is well documented, and this knowledge led to several clinical trials based on the modulation of the adaptive autoimmune response. However, for most AiDs, these clinical trials were unsatisfactory, with limited success and few definitive cures (1, 2). In parallel, during the last decades, the role of innate immunity in the immunopathology of AiDs has emerged, and it is tempting to speculate that targeting this innate part of the immune system may be a promising therapeutic approach against AiDs (3). Among the numerous molecules produced by the innate immune system, the antimicrobial peptides (AMPs) have recently been identified as important factors in the development of AiDs. Importantly, the role of AMPs in the autoimmune process appears to be complex, with both a deleterious and a protective role. Originally discovered in silk moth 40 years ago, it is now recognized that AMPs, also known as host defense peptides, represent a major component of the innate immune system of every living organism (4). AMPs are a large group of small cationic polypeptide molecules largely produced at the epithelial surfaces, but not exclusively, and their first described function is to protect against the continuous exposure to environmental microorganisms (5); more recently, their antimicrobial function extends to the maintenance of the host microbiota (6). AMPs encompass representatives of several distinct molecules including cathelicidins, alpha-defensins (human neutrophil peptides, HNPs), beta-defensins (BDs), regenerating islet-derived protein, ribonucleases, or S100 proteins. These polypeptides exert their antimicrobial activity by directly disrupting the membrane of microorganisms or by the sequestration of metals essential for microorganism growth, for example (7). Importantly, high concentrations of AMPs may be toxic for eukaryotic host cells which support their use as anticancer drugs (8). More recently, AMPs were shown to directly modulate the function of non-immune cells; for example, AMPs regulate the intestinal barrier integrity by stimulating tight junction protein synthesis by enterocytes (9) or promoting insulin secretion by pancreatic beta cells (10). Importantly, AMPs have been rediscovered in the last decades as an important player in the regulation of the immune responses, which supports their use as potential therapeutic molecules against immune-related diseases (8, 11). Here, we will discuss the role of AMPs in several AiDs and attempt to propose some hypothesis regarding their contrasting role in autoimmunity. The increasing knowledge about the role of AMPs in autoimmunity may open new therapeutic opportunity to prevent or cure AiDs.

## Antimicrobial Peptides as Immunomodulatory Molecules

During the last decades, the ability of AMPs to act as modulators of the immune response has been extensively studied, and their role in innate and adaptive immunity has become increasingly appreciated (8, 12). The immunomodulatory roles of cathelicidin and defensins have been extensively investigated as these AMPs are expressed in various cell types, including epithelial cells and cells of the immune system (13). First of all, it is important to mention that the impact of AMPs on the immune system is widespread and complex, with both pro- and anti-inflammatory effects, likely reflecting the necessity of a tight control of any immune responses. Due to their ability to bind chemokine receptors, cathelicidins and defensins are potent chemoattractants for several immune cell types, including monocytes *via* CCR2, neutrophils *via* formyl-peptide receptors, dendritic cells (DCs), and T cells *via* G protein-coupled receptors (14–16). However, the immune cells recruited by AMPs can potentially have either inflammatory or regulatory functions, and the chemotactic activity of AMPs cannot be necessarily associated with inflammation. The presence of AMPs during the differentiation of macrophages and DCs can bias their polarization toward a pro-inflammatory phenotype (17–20). However, one study also demonstrated the role of HNP1–3 on human monocyte-derived DCs showing that, depending on the dose of HNPs, they can either promote at a low dose or prevent at a high dose the differentiation and maturation of DCs (21). AMPs also modulate the activation of macrophages and DCs through their capacity to bind Toll-like receptor (TLR) ligands. By sequestrating TLR ligands or perturbating intracellular signaling pathways, AMPs inhibit the activation of macrophages and DCs. After being endocytosed in monocytes, LL-37 binds to GAPDH, and the resulting complex interacts with p38 MAPK and other signaling molecules to prevent excessive inflammation (12, 22). However, AMPs also have an adjuvant role by enhancing the pro-inflammatory response to TLR ligands such as viral RNA *via* TLR3 in epithelial cells, flagellin *via* TLR5 in keratinocytes, and CpG *via* TLR9 in B cells and plasmacytoid DCs (pDCs) (23–25). Finally, AMPs regulate the apoptosis of innate immune cell types as neutrophils prolonging their life after activation (26, 27). The above-mentioned effects of AMPs on innate immune cells, and particularly on antigen-presenting cells, impact the adaptive immune response by modulating Th1, Th17, or regulatory T (Treg) cell responses (18, 28, 29). In summary, AMPs occupy a central place not only in the innate immune defense against invading pathogens but also in the modulation of the adaptive immune response. AMPs may be required to initiate a fast immune response and then to efficiently terminate the response and prevent immune-induced tissue damage. Consequently, a dysregulated expression of AMPs in a specific tissue may participate in the development of the autoimmune response, as described in the following sections. Besides, research during the last decade has revealed the significant role of the microbiota in the regulation of autoimmune diseases (30). Consequently, thanks to the ability of AMPs to regulate microbiota composition (6), they likely also modulate the autoimmune response in this way.

## The Role of AMPs in Autoimmune Disease

From primary observations that the expression of AMPs is dysregulated in many tissues affected by autoimmune or autoinflammatory diseases, their involvement in the pathophysiology of these diseases is now established or suspected, as in systemic lupus erythematosus (SLE), psoriasis, rheumatoid arthritis (RA), type 1 diabetes (T1D), Sjögren’s disease (SjS), and multiple sclerosis (MS). The most documented aspect is that an aberrant production of AMPs produced by neutrophils or epithelial cells promotes inflammation, favoring the autoimmune response (31). Activated neutrophils in the tissue produce neutrophil extracellular traps (NETs) that are made of self-nucleic acids from the nucleus bound to granular cytoplasmic proteins rich in AMPs (32). These NETs are normally produced in infectious context to immobilize and kill pathogens (33, 34). Aberrant production of NETs in sterile condition and impaired clearance of these NETs would stimulate pDCs *via* TLR7 and TLR9 to produce type I interferons (IFNs), which are important contributors to autoimmune diseases by activating antigen presentation by DCs and the production of autoantibodies by B cells (35–39). On the other hand, recent studies have shown that AMPs produced by specific non-immune cells carry immunoregulatory properties on various innate and adaptive immune cell types, leading to the induction of Treg cells, preventing the development of autoimmune disease (40). In the present review, we discuss the present knowledge about the role of AMPs in autoimmune diseases.

### Systemic Lupus Erythematosus

Systemic lupus erythematosus is a systemic autoimmune disease that results from defects of the immune system that can occur at different levels of the immune response, explaining the vast heterogeneity of the clinical presentation of the disease. Affected tissues include the central nervous system (CNS), kidney, blood, skin, and joints (41). SLE is a disease caused by an inappropriate reaction of the innate and adaptive immune systems and is characterized by the presence of autoantibodies to nuclear antigens forming immune complexes with DNA or RNA. SLE is also characterized by a type I IFN signature that results from the sterile activation of pDCs by the immune complexes (42). Both HNPs and cathelicidin have been implicated in the physiopathology of SLE (43). Increased levels of HNP1–3 expressed by activated neutrophils have been detected by enzyme-linked immunosorbent assay (ELISA) in the blood of SLE patients (44–46). These HNPs harbor chemotactic and pro-inflammatory activity for immune cells such as DCs and T cells (47). More importantly, autoantibodies against HNPs are detected in the sera of SLE patients, and the HNP level correlates with disease activity (48). Using antibody suspension bead array, Idborg et al. determined that the level of S100 calcium-binding protein A12 was increased in the serum of patients compared with healthy individuals (49). Higher levels of cathelicidin have been observed by *in situ* hybridization in the skin of SLE patients compared with healthy individuals (50). However, the serum level of LL-37 measured by ELISA did not increase in patients vs. healthy individuals and did not correlate with disease activity in patients (51). Gilliet’s group described the pathogenic behavior of cathelicidin in SLE. The pathogenic role of cathelicidin in SLE originates from its presence in NETs and its ability to form and stabilize immune complexes with DNA and autoantibodies. As described above, these complexes promote type I IFN secretion by pDCs and autoantibody production by B cells (52, 53). Recently, another aspect of the role of cathelicidin in SLE has been identified. The authors show that cathelicidin-specific T cells circulate in patients and support the production of cathelicidin-specific pathogenic autoantibodies by B cells (54). Animal models of lupus also demonstrated the role of AMPs in the physiopathology of the disease. In the New Zealand mixed (NZM) model, the accumulation of NETs and autoantibodies against the NET component including cathelicidin have been reported (55). However, using a model of pristane-induced lupus, cathelicidin-related antimicrobial peptide (CRAMP)-deficient mice were not protected against the disease, minimizing the causative role of cathelicidin in lupus (56).

### Psoriasis

Psoriasis is an autoimmune disease affecting mainly the skin with the presence of inflammatory plaques for the most common form. The immune pathogenesis of psoriasis implicates dysfunction of the innate and adaptive immunity with the recruitment of inflammatory macrophages and type I IFN-producing pDCs and the generation of an uncontrolled Th17 response (57). By reverse transcription PCR (RT-PCR) and immunohistochemistry, cathelicidin and human beta-defensins 2 and 3 (hBD2/3) have been shown to be highly expressed in the psoriatic skin of patients (58–63). Gilliet’s group elegantly deciphers the pathogenic role of cathelicidin in psoriasis. As described above, cathelicidin binds to self-DNA/RNA released from keratinocytes to form immunogenic complexes that activate type I IFN-secreting pDCs through TLR9/TLR7 (25, 64). Moreover, cathelicidin-immune complexes activate 6-sulfo LacNAc (slan) DCs *via* TLR7/8 that, in response, secrete inflammatory cytokines [interleukin (IL)-6, IL-12, and IL-23], inducing Th1/Th17 responses (65). A recent study also demonstrates the role of cathelicidin from infiltrating neutrophils in the disease. Complexes of cathelicidin with RNA that are rich in psoriatic skin trigger *via* TLR8/TLR13 inflammatory cytokine production by neutrophils and the formation of NETs perpetuating chronic inflammation in psoriasis (66). In addition to activating the innate immune system, cathelicidin was identified as an autoantigen with the presence of cathelicidin-specific T cells that produce IFN-gamma in the skin of patients with psoriasis (67). Also, circulating autoantibodies to cathelicidin and its citrullinated or carbamylated derivatives were found in psoriasis patients. However, their role in the pathogenesis of the disease remains to be determined (68, 69).

### Rheumatoid Arthritis

Rheumatoid arthritis is a chronic inflammatory disease of the joints resulting in cartilage and bone damage (70). The synovial fluid of RA patients is infiltrated by innate immune cells (e.g., monocytes, DCs, mast cells, and innate lymphoid cells) and adaptive immune cells (e.g., Th1 and Th17 cells and B cells). While RA is pathologically heterogeneous, more severe symptoms are associated with the presence of autoantibodies against posttranslationally modified self-peptides, especially from proteins that have been citrullinated or carbamylated (71). Different AMPs are expressed constitutively or are inducible in articular joints such as hBD1–3 and cathelicidin (72). Proteomic analysis and ELISA revealed that, in patients, HNP1–3 expressions are also increased in the synovia of patients with an observed correlation between joint erosion and the HNP levels (73, 74). The presence of pro-inflammatory cytokines in the diseased joints may likely explain the increased expressions of some AMPs, such as hBD2/3 (72, 75). hBD3 may participate in the physiopathology of RA since this AMP stimulates the production of metalloproteinases by chondrocytes, degrading the extracellular matrix of cartilage (72, 75). Besides, hBDs are also known as potent chemotactic agents for human monocytes, dendritic cells, and T cells (47). Increased expressions of hBD2/3 may also contribute to recruiting immune cells and amplifying inflammatory response in the joint. Increased expression of cathelicidin has been described in the synovia of RA patients, with macrophages and neutrophils as cell sources identified by flow cytometry (76, 77), and cathelicidin induces the apoptosis of osteoblasts, indirectly contributing to altered bone formation in arthritic joints (78). Finally, using the pristane-induced arthritis model in rats, Hoffmann et al. have demonstrated that cathelicidin is produced by neutrophils in the synovial fluids of diseased rats and that the transfer of pristane-primed neutrophils induced arthritis, whereas type I IFNs or autoantibody responses in control rats did not (77). Altogether, exaggerated cathelicidin expression in the joints may participate in the development of RA; however, the exact pathogenic mechanism remains unclear. It could be hypothesized that cathelicidin from neutrophils may prime pDCs to secrete type I IFNs, as demonstrated for other autoimmune diseases (38). Interesting in the context of psoriatic arthritis, posttranslationally modified cathelicidin from neutrophils represents a source of self-antigens, supporting that autoantibodies against cathelicidin participate in inflammation and the autoimmune process (79).

### Type 1 Diabetes

Type 1 diabetes is an autoimmune disease ultimately resulting from the destruction of the insulin-producing β-cells of the pancreas by autoreactive T cells. However, many different innate and adaptive immune cell types are implicated in the long diabetogenic process. Due to the inability to produce insulin, T1D patients are unable to control their glycemia, and even with replacement therapy, i.e., insulin injection, they can develop diabetes-associated complications in multiple organs (80). Few studies have examined the expressions of AMPs in T1D patients. By ELISA, Brauner et al. described reduced levels of cathelicidin and hBD1 in the serum of T1D patients compared with type 2 diabetic patients or healthy individuals (81). Besides, Nemeth et al. showed by ELISA and reverse transcription quantitative PCR (RT-qPCR) increased levels of HNP1–3 in the plasma of T1D patients; however, similar increases were observed in type 2 diabetes (T2D) patients, suggesting that hyperglycemia may be responsible for such increases and, consequently, may only be a consequence of the disease and not a cause (82). Indeed, hyperglycemia was demonstrated in a diabetic rat model to promote NET formation (83). Importantly, these studies measured the circulating levels of AMPs in patients that may not reflect the levels in the pancreas. Moreover, the highest concentrations of HNPs were detected in T1D patients with complications including diabetic kidney disease (82). The explanation might be that the elevations in the plasma HNP1–3 levels are the consequence of the decreased renal degradation of the peptides in patients with advanced nephropathy (84). The role of cathelicidin in T1D development has been well demonstrated in a non-obese diabetic (NOD) mouse model. A first study from our group demonstrated that cathelicidin participates in the initiation of the disease in young NOD mice (85). Around the age of weaning, netting neutrophils transiently infiltrate the pancreas and produce cathelicidin in complex with self-DNA and anti-DNA immunoglobulin G (IgG). These complexes activate pDCs *via* TLR9, inducing the production of type I IFNs that promotes the progression of T1D (86). Importantly, a similar mechanism may be at play in human since aberrant neutrophil activation in the blood and the presence of NETs in the pancreatic section have been identified in pre-diabetic and diabetic patients (87, 88). In addition, in a follow-up study, we have demonstrated the protective role of cathelicidin against the disease. Indeed, we identified that cathelicidin is normally produced by pancreatic β-cells in adult non-autoimmune mice, but not in NOD mice. Conversely, treatment of pre-diabetic adult NOD mice with recombinant cathelicidin induces regulatory macrophages and T cells in the pancreas, preventing the development of the disease. We demonstrated that the gut microbiota-derived metabolites short-chain fatty acids (SCFAs) promote the pancreatic production of cathelicidin, and the alteration in the gut microbiota explains the defective production of cathelicidin in NOD mice (89). The protective effect of SCFAs against T1D has been demonstrated by others in mouse models (90) and in patients (91). How the same AMP, cathelicidin, has apparent opposite effects in T1D is under investigation by our group. Finally, we recently demonstrated that the pancreatic β-cells also produce mouse β-defensin 14 (mBD14) under the control of the gut microbiota. This expression of mBD14 in the pancreas is defective in the NOD mice compared with the non-autoimmune mouse strains, and treatment of pre-diabetic NOD mice with recombinant mBD14 prevents diabetes development by the induction of regulatory B cells in the pancreas (92). Overall, the pancreatic β-cells harbor the capacity to produce different immunoregulatory AMPs targeting different immune cell types, ensuring the maintenance of the immune tolerance in the pancreas. Defective AMP expression by the pancreatic β-cells allows the inflammation to develop in the pancreas, favoring the diabetogenic autoimmune adaptive response. However, cathelicidin aberrantly expressed by neutrophils infiltrating the pancreas in a diabetes-prone genetic background participates in the initiation of the disease *via* a classical mechanism described for other autoimmune diseases.

### Sjögren’s Syndrome

Sjögren’s syndrome is a chronic autoimmune disease affecting primarily the exocrine glands; hallmarks of the disease associate with dry mouth (xerostomia) and dry eyes (keratoconjunctivitis sicca). Moreover, multiple organs can be affected, including the lung, kidney, liver, joint, skin, and so on. The impairment of the salivary and lacrimal glands (SGs and LGs, respectively) is caused by the infiltration of various immune cell types, including T and B cells, macrophages, and DCs (93). In addition, SjS diagnosis relies on the presence of autoantibodies against ribonucleoproteins, type I IFN production by infiltrating pDCs, and actually many features of SjS are indeed in common with other systemic autoimmune diseases (94–96). The literature regarding the potential role of AMPs in SjS is not abundant; however, some studies suggest that cathelicidin and defensins may have a role in the physiopathology of the disease. Cathelicidin expression is detected by RT-qPCR and immunohistochemistry in both mouse and human SGs at steady state, and cathelicidin expression is upregulated with inflammation of the oral cavity (97, 98). Svensson et al. also reported that cathelicidin of the parotid and submandibular/sublingual saliva originates from the glandular blood vessel neutrophils (99). Besides, patients with morbus Kostmann have congenital neutropenia; neutrophils from these patients were deficient in LL-37, and no cathelicidin is detected by mass spectrometry and Western blot in the plasma and the whole saliva of these patients, suggesting that salivary LL-37 is indeed derived from neutrophils (100). The reports above support that chronic inflammation may be responsible for the increase of cathelicidin expression observed in SGs in the context of SjS. hBD1–3 mRNAs have been detected in SGs, including the parotid, submandibular, and minor glands, as well as the oral epithelium (101, 102). One study confirmed the expressions of hBD1–3 in SGs and showed by immunohistochemistry that the expressions of hBD1/2 were decreased in minor SGs from SjS patients compared with healthy subjects (103). By proteomic analysis, HNP1 expression was found specifically upregulated in the SGs of SjS patients, together with other inflammatory genes (104, 105). Unlike cathelicidin, hBD1/2 in SGs may largely derive from ductal epithelial cells, which may explain the decreased hBD levels as a consequence of the destruction of ductal epithelial cells during inflammation. Our laboratory is currently investigating how these AMPs may participate in the development of SjS. Regarding the above literature, it is tempting to speculate that cathelicidin or HNP-forming immune complexes may trigger type I IFN production by pDCs infiltrating the SGs; however, we could also hypothesize that cathelicidin or BDs are able to maintain immune tolerance in the SGs by inducing immunoregulatory immune cells.

### Multiple Sclerosis

Multiple sclerosis is a chronic inflammatory disorder of the CNS. Hallmarks of the disease associate with multifocal demyelination, axonal loss, activation of glial cells, infiltration by innate and adaptive immune cells, and the presence of autoantibodies, together initiating the demyelination of axons (106, 107). AMPs appear to be part of the CNS immune system as defensins and cathelicidins are produced by a variety of cell types in the brain such as astrocytes and microglia (108, 109). Using RT-qPCR and Western blot, AMPs have been detected in the CNS of rodents and humans at steady state and in inflammatory conditions (109–113). Accumulating evidences support that infiltrating neutrophils may play an important role in the diseases affecting the CNS (114), including MS (115); however, whether these neutrophils express cathelicidin remains unknown. Using the experimental autoimmune encephalomyelitis mouse model of MS, one study showed that recombinant mBD14 has a protective and even a therapeutic effect against the disease by directly stimulating Treg cells (116). Lastly, a study has shown that a parasitic cathelicidin-like peptide is protective against both T1D and MS in mouse models (117). Although more studies are required to support the role of AMPs in MS, AMPs may represent interesting therapeutic tools against MS.

## Conclusion

**FIGURE 1:**
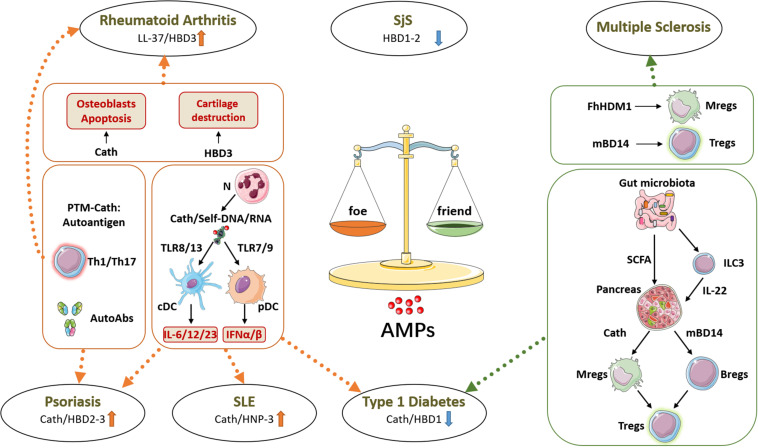
Friend or foe: roles of antimicrobial peptides (AMPs) in autoimmune diseases. AMPs have a dysregulated expression in many tissues affected by autoimmune diseases, where they can promote or prevent the autoimmune response. On one side, as shown in psoriasis, systemic lupus erythematosus (SLE), and type 1 diabetes (T1D), cathelicidin (Cath) produced by neutrophils (N) forms immune complexes with self-nucleic acids, activating conventional and plasmacytoid dendritic cells (cDCs and pDCs, respectively) to release inflammatory cytokines which boost the autoimmune response. Besides, in psoriasis and in rheumatoid arthritis, posttranslational modifications of AMPs can generate neo-self-antigens recognized by effector T cells (Th1/Th17) and produce autoantibodies (AutoAbs). Moreover, in rheumatoid arthritis, AMPs directly support cartilage destruction by acting on osteoblasts and osteoclasts. On the other side, AMPs can also prevent autoimmune diseases, as shown in T1D. The gut microbiota-derived metabolites short-chain fatty acids (SCFAs) can induce AMP expression in pancreatic islets, and these AMPs directly or indirectly induce protective and regulatory immune cells including macrophages (Mregs), B cells (Bregs), and T cells (Tregs). In multiple sclerosis, mBD14 and *Fasciola hepatica* helminth defense molecule 1 (FhHDM1) show similar immunoregulatory functions and protective effects in a mouse model of the disease. h, human; m, mouse, BD, beta-defensin; HNP, alpha-defensin; SjS, Sjögren syndrome.

Since their discovery 50 years ago as microbicidal molecules, AMPs appear today as key molecules in the regulation of the immune responses, and not surprisingly, the dysregulation of their expression participates in the development of various autoimmune diseases. However, the precise role of AMPs in autoimmunity seems complex, with both detrimental and protective effects even considering the same AMP and the same disease, such as cathelicidin in T1D (Figure 1). Understanding the opposite role of AMPs in autoimmune diseases is a crucial step before the development of new therapeutic strategies based on AMPs for resolving the progression of these diseases. AMPs are chemoattractants for various immune cells; however, the phenotype of these cells can be either inflammatory or regulatory. Overall, cathelicidin produced by neutrophils appears to be a potent inducer of type 1 IFNs and inflammatory cytokines favoring the development of the autoimmune responses. Recent studies also support that cathelicidin and HNPs from neutrophils are a source of autoantigens. On the other side, secretion of cathelicidin and BDs by the cells targeted by the autoimmune attack may represent a mechanism of protection *via* the induction of regulatory immune cells. One attractive hypothesis to explain the dual role of AMPs in AiDs is that the immune function of AMPs is related to posttranslational modifications of peptides, such as citrullination or carbamylation. Indeed, such modifications of cathelicidin reduce its positive charge, increase its chemotactic activity, and alter its ability to bind nucleic acids, thereby reducing their pro-inflammatory potential (118, 119). Also, modified AMPs may represent a source of autoantigens, but not their native forms. Importantly, modifications of susceptible proteins that occur in inflammatory conditions such as in activated neutrophils may represent a general mechanism of control of the inflammatory response. Whether this mechanism of innate immune tolerance is defective in the autoimmune context remains to be determined. Considering the growing knowledge about the role of AMPs in AiDs, it is tempting to suggest their use as therapeutic targets or agents to prevent or treat AiDs (120). However, due to their conflicting and pleiotropic immunomodulatory roles, the use of AMPs should be considered with care. Unexpectedly, a safer and efficient AMP-based therapy against AiDs may take advantage of their ability to shape the microbiota. Using this skill, AMPs may correct the pathological microbiota prevailing in autoimmune-prone individuals, hence preventing the development of AiDs.

## Author Contributions

WL and JD reviewed the literature, wrote the manuscript, and designed the figure.

## Conflict of Interest

The authors declare that the research was conducted in the absence of any commercial or financial relationships that could be construed as a potential conflict of interest.
